# Causal roles of skin and gut microbiota in skin appendage disorders suggested by genetic study

**DOI:** 10.3389/fimmu.2024.1427276

**Published:** 2024-09-10

**Authors:** Yuhang Zhu, Wanguo Liu, Mei Wang, Xu Wang, Sibo Wang

**Affiliations:** ^1^ Department of Orthopedics, China-Japan Union Hospital of Jilin University, Changchun, China; ^2^ Department of Dermatology, The First Hospital of Jilin University, Changchun, China; ^3^ Department of Neurology, Center for Neuroscience, The First Hospital of Jilin University, Changchun, China

**Keywords:** skin microbiota, gut microbiota, skin appendage disorders, Mendelian randomization, causal inference

## Abstract

**Objectives:**

There is evidence from observational studies that human microbiota is linked to skin appendage Disorders (SADs). Nevertheless, the causal association between microbiota and SADs is yet to be fully clarified.

**Methods:**

A comprehensive two-sample Mendelian randomization (MR) was first performed to determine the causal effect of skin and gut microbiota on SADs. A total of 294 skin taxa and 211 gut taxa based on phylum, class, order, family, genus, and ASV level information were identified. Summary data of SADs and eight subtypes (acne vulgaris, hidradenitis suppurativa, alopecia areata, rogenic alopecia, rosacea, rhinophyma, seborrhoeic dermatitis, and pilonidal cyst) were obtained from the FinnGen consortium. We performed bidirectional MR to determine whether the skin and gut microbiota are causally associated with multiple SADs. Furthermore, sensitivity analysis was conducted to examine horizontal pleiotropy and heterogeneity.

**Results:**

A total of 65 and 161 causal relationships between genetic liability in the skin and gut microbiota with SADs were identified, respectively. Among these, we separately found 5 and 11 strong causal associations that passed Bonferroni correction in the skin and gut microbiota with SADs. Several skin bacteria, such as *Staphylococcus*, *Streptococcus*, and *Propionibacterium*, were considered associated with multiple SADs. As gut probiotics, *Bifidobacteria* and *Lactobacilli* were associated with a protective effect on SAD risk. There was no significant heterogeneity in instrumental variables or horizontal pleiotropy.

**Conclusions:**

Our MR analysis unveiled bidirectional causal relationships between SADs and the gut and skin microbiota, and had the potential to offer novel perspectives on the mechanistic of microbiota-facilitated dermatosis.

## Introduction

As the largest organ of the body, the skin functions as our primary barrier against external threats. However, it also serves as a diverse environment for a multitude of microorganisms, whose interactions significantly contribute to the skin’s overall health, immune response, and disease development (1). Skin appendages, including sweat glands, hair follicles, sebaceous glands, and arrector pili muscles, are found in the dermis and adipose tissue. The surface and appendages of the skin are colonized by the skin microbiota, whose composition is contingent upon the microenvironment (2). Disturbances in the balance of the skin microflora, known as dysbiosis, have been recognized as contributing factors in various dermatological conditions, especially skin appendage disorders (SADs) (3, 4).

Meanwhile, the gut, home to the densest microbial population within the human body, exerts effects that extend beyond its confines. Gut microorganisms are primarily recognized for their roles in metabolic processes and immune system development (5). Interestingly, their influence transcends the gut, affecting various physiological systems, including the skin (6). This influential link between the gut and skin, termed the gut-skin axis, is emerging as an integral component in skin health and disease (6, 7). Despite these advances, the concurrent role of gut and skin microbiota in SADs remains an intriguingly uncharted area of research. Additionally, without experimental methods that rely on cultivated isolates, it is challenging to determine causality due to the close relationship between the microbiota and its host (8, 9).

Mendelian randomization (MR) is a powerful epidemiological technique that indicates causal associations by utilizing genetic variations (10). MR is inherently not confounding since environmental and self-adapted variables have no effect on genetic differences, as these are randomly allocated at conception. Moreover, this approach can circumvent the issue of reverse causality, as germline genotypes remain unaltered by physiological disturbances resulting from disease. Our study embarks on an exploration using comprehensive bidirectional MR to unravel potential causal relationships between the skin and gut microbiota and multiple SADs (acne vulgaris, hidradenitis suppurativa, alopecia areata, androgenic alopecia, rosacea, rhinophyma, seborrheic dermatitis, and pilonidal cyst). We aim to offer insights into the possible involvement of these microbial communities in the pathogenesis and progression of SADs, and pave the way for microbiome-oriented therapeutic strategies.

## Methods

### Data sources

Genetic variations of skin microbiota were derived from the GWAS conducted by Moitinho-Silva et al. (11). A sum of 1656 skin samples was acquired from individuals within two German cohorts, KORA FF4 (n = 635) and PopGen (n=1021). The samples were collected from three skin microenvironments, including moist skin (antecubital fossa in both cohorts), dry skin (dorsal and volar forearm in PopGen), and sebaceous skin (forehead in PopGen and retroauricular fold in KORA FF4). Microbial community patterns were obtained through the 16 S rRNA gene. Amplicon sequence variants (ASVs) and taxonomic groups from genus to phylum level were utilized in the GWAS. In total, 294 taxa in both cohorts were included in the analysis (14 phyla, 22 classes, 24 orders, 30 families, 54 genera, and 150 ASVs).

SNPs associated with the composition of the gut microbiota were selected as instrumental variables (IVs) within a GWAS database belonging to the MiBio-Gen consortium (12). This large-scale multi-ethnic GWAS integrated 16S rRNA gene sequencing data from 18,340 individuals across 24 cohorts to investigate the link between human autosomal genetic variants and the intestinal microbiota. There were 211 taxa in all, including 9 phyla, 16 classes, 20 orders, 35 families, and 131 genera.

GWAS summary data for SADs (377,277 individuals), acne vulgaris (AV) (363,927 individuals), hidradenitis suppurativa (HS) (362,071 individuals), alopecia areata (AA) (361,822 individuals), androgenic alopecia (AGA) (201,214 individuals), rosacea (ROS) (363,350 individuals), rhinophyma (RPH) (361,275 individuals), seborrhoeic dermatitis (SD) (339,277 individuals), and pilonidal cyst (PC) (358,708 individuals) were acquired from the R9 release of FinnGen consortium (13). Comprehensive information regarding the encompassed cohorts, genotypic data, endpoint specifications, and association testing can be accessed through the FinnGen webpage.

### Instrumental variable selection

We applied the following criteria to select the instrumental variables (IVs): (1) potential IVs were identified as single nucleotide polymorphisms (SNPs) at the locus-wide significance threshold (*P* < 1.0×10^–5^) that was widely utilized in the previous MR studies (14–17); (2) a linkage disequilibrium parameter (*R* ^2^) of SNP was set at 0.01, with a genetic distance of 10,000 kb; (3) A minor allele frequency (MAF) of less than 0.01 was used to eliminate SNPs; (4) palindromic SNPs were discarded to guarantee that the allelic effects of SNPs during the harmonization process; and (5) IVs with an *F* statistic <10 were excluded.

### Mendelian randomization analysis

**Figure 1:**
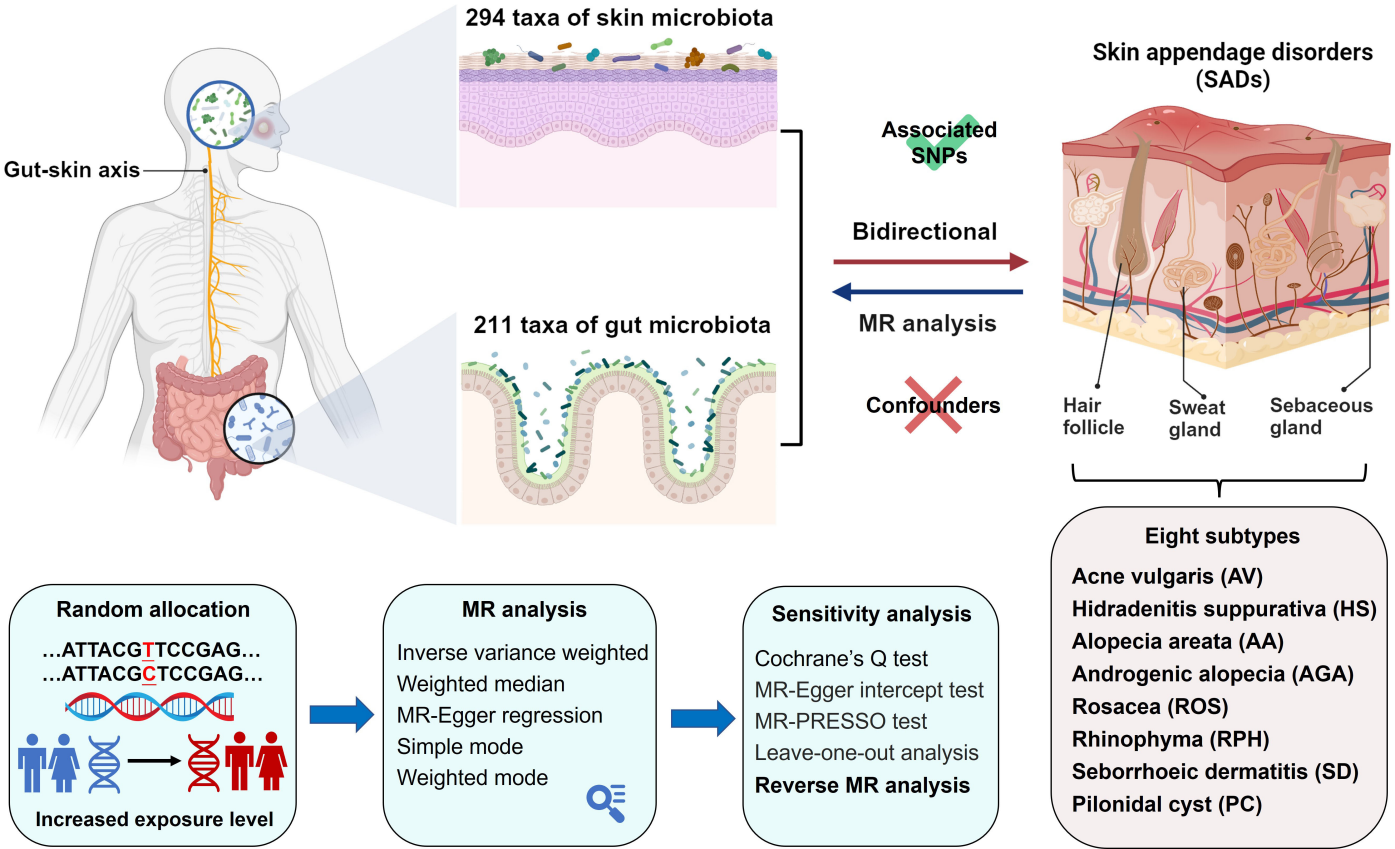
Study design and flowchart.

The MR study was structured as depicted in **Figure 1**. We applied five MR methods for features with multiple IVs: inverse-variance weighted (IVW) (18), weighted median (19), MR-Egger regression (20), simple mode (21), and weighted mode (22). The IVW method has been shown to have more power than the others under some conditions (22); hence, we mainly used the IVW method for the results, and the other four methods as supplements. For a more stringent interpretation of the causal relationship, we performed a Bonferroni correction, based on the number of bacteria within each level. For skin microbiota, the threshold significance was set as follows: phylum *P* = 7.14 × 10^−3^ (0.05/7), class *P* = 4.55 × 10^−3^ (0.05/11), order *P* = 4.17 × 10^−3^ (0.05/12), family *P* = 3.33 × 10^−3^ (0.05/15), genus *P*= 1.85 × 10^−3^ (0.05/27), ASV *P*= 6.67 × 10^−4^ (0.05/75). For gut microbiota, the threshold significance was set as follows: phylum *P* = 5.56 × 10^−3^ (0.05/9), class *P* = 3.13 × 10^−3^ (0.05/16), order *P* = 2.50 × 10^−3^ (0.05/20), family *P* = 1.43 × 10^−3^ (0.05/35), genus *P*= 3.82 × 10^−3^ (0.05/131). *P*-values falling within the range between 0.05 and the corrected value were regarded as nominal significance with potential causal effects. To investigate whether SADs exerted any causal influence on the identified skin and gut microbiota, we also conducted a reverse MR analysis. The methodologies and settings employed were in line with those of the forward MR. Two-sample MR (version 0.5.6) and MRPRESSO (version 1.0) packages with R software (version 4.2.2) were used. The MR study was conducted in accordance with STROBE-MR guidelines (23, 24).

### Sensitivity analysis

To evaluate the heterogeneity of instrumental variables, we employed Cochran’s Q statistics (25). Additionally, a leave-one-out (LOO) analysis was conducted to assess the influence of individual SNPs on the overall causal estimate (26). By systematically excluding each SNP from the analysis, we evaluated the robustness of our results. Consistent results across LOO analyses enhance confidence in the causal inference, indicating that the findings are not driven by specific SNPs (26). We also used MR-Egger intercept tests and MR-PRESSO to verify the existence of horizontal pleiotropy. MR-PRESSO performs a global test to detect the presence of horizontal pleiotropy by comparing the observed distribution of SNP-exposure and SNP-outcome associations with their expected distribution under no pleiotropy (27). A significance (*P* < 0.05) indicates a substantial influence of pleiotropic SNPs on the original causal estimate. In MR-Egger regression, the intercept term provides an estimate of the average pleiotropic effect across all SNPs (28). A non-zero intercept indicates the presence of directional pleiotropy. To further confirm if the observed causalities were skewed due to reversed causation, the Steiger directionality test was applied (29). If the SNPs explain more variance in the exposure than in the outcome, it supports the correct direction of causality (27). Confirming the direction of effect reduces the risk of reverse causation bias, where the outcome could mistakenly appear to cause the exposure (27). Given the distinctiveness of skin microbiota across different microenvironments (moist, dry, and sebaceous areas) in KORA FF4 and PopGen cohorts, meta-analyses were carried out by combining data sets originating from the same microenvironment. Statistical analyses were applied using the META (version 6.5.0) package.

## Results

### SNP selection

Following the quality control procedures, a total of 838 SNPs and 1031 SNPs in the forward MR analysis were selected as IVs from skin and gut microbiota, respectively (**Supplementary Tables S1, S2**). In the reverse MR analysis, a total of 3316 SNPs and 3259 SNPs were severally selected from SADs as IVs for skin and gut microbiota (**Supplementary Tables S3, S4**). The F statistics of the remaining IVs were all greater than 10, which suggests that there was less chance of weak instrument bias affecting the estimates. Detailed information on the major IVs in the MR analysis that passed the Bonferroni correction between SADs and skin and gut microbiota was displayed in **Supplementary Table S5**.

### Causal associations of skin and gut microbiota and SADs

**Figure 2:**
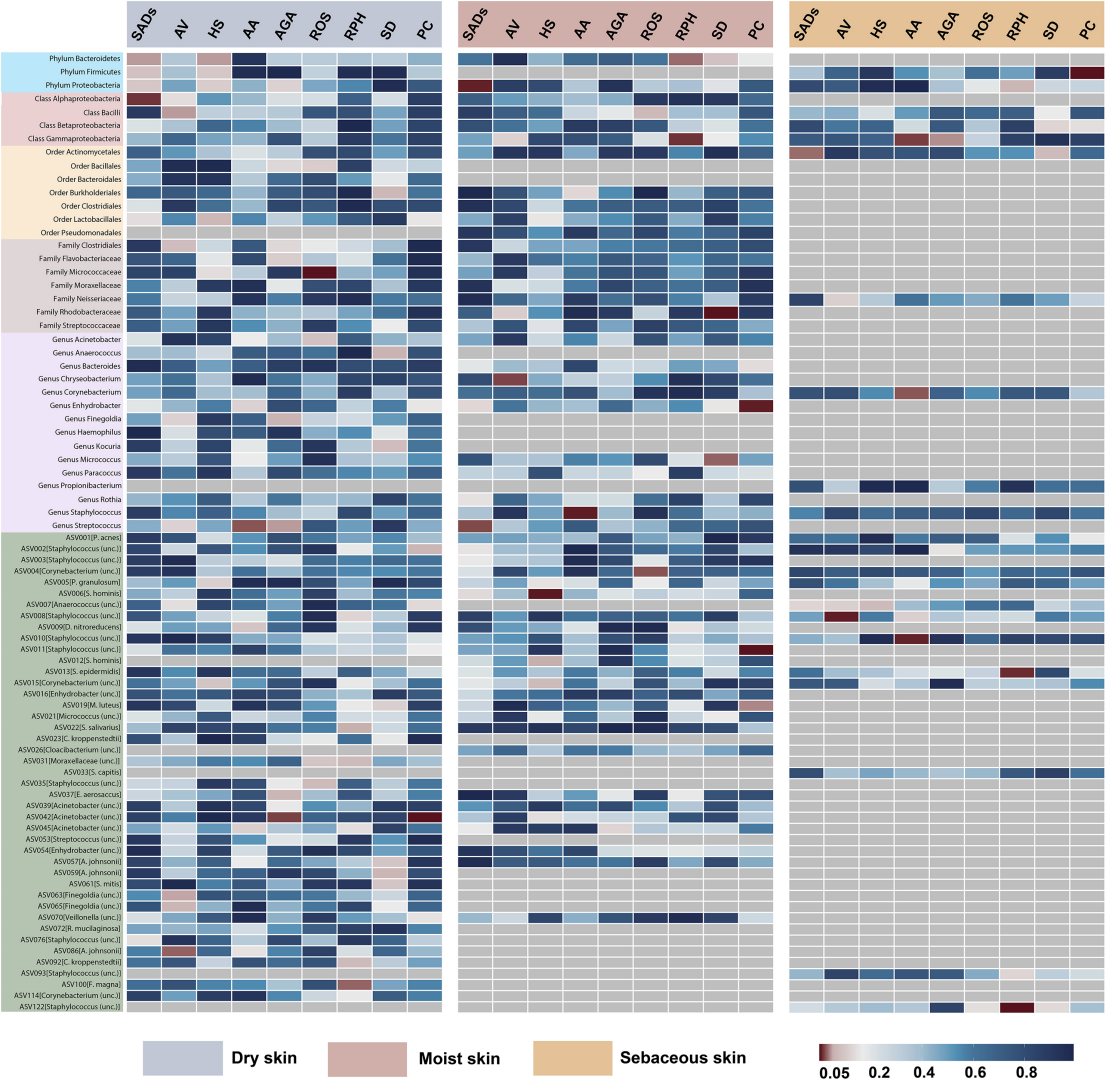
Heatmap showing causal associations between skin microbiota and SADs. SADs, skin appendage Disorders; AV, acne vulgaris; HS, hidradenitis suppurativa; AA, alopecia areata; AGA, androgenic alopecia; ROS, rosacea; RPH, rhinophyma; SD, seborrhoeic dermatitis; and PC, pilonidal cyst.

**Figure 3:**
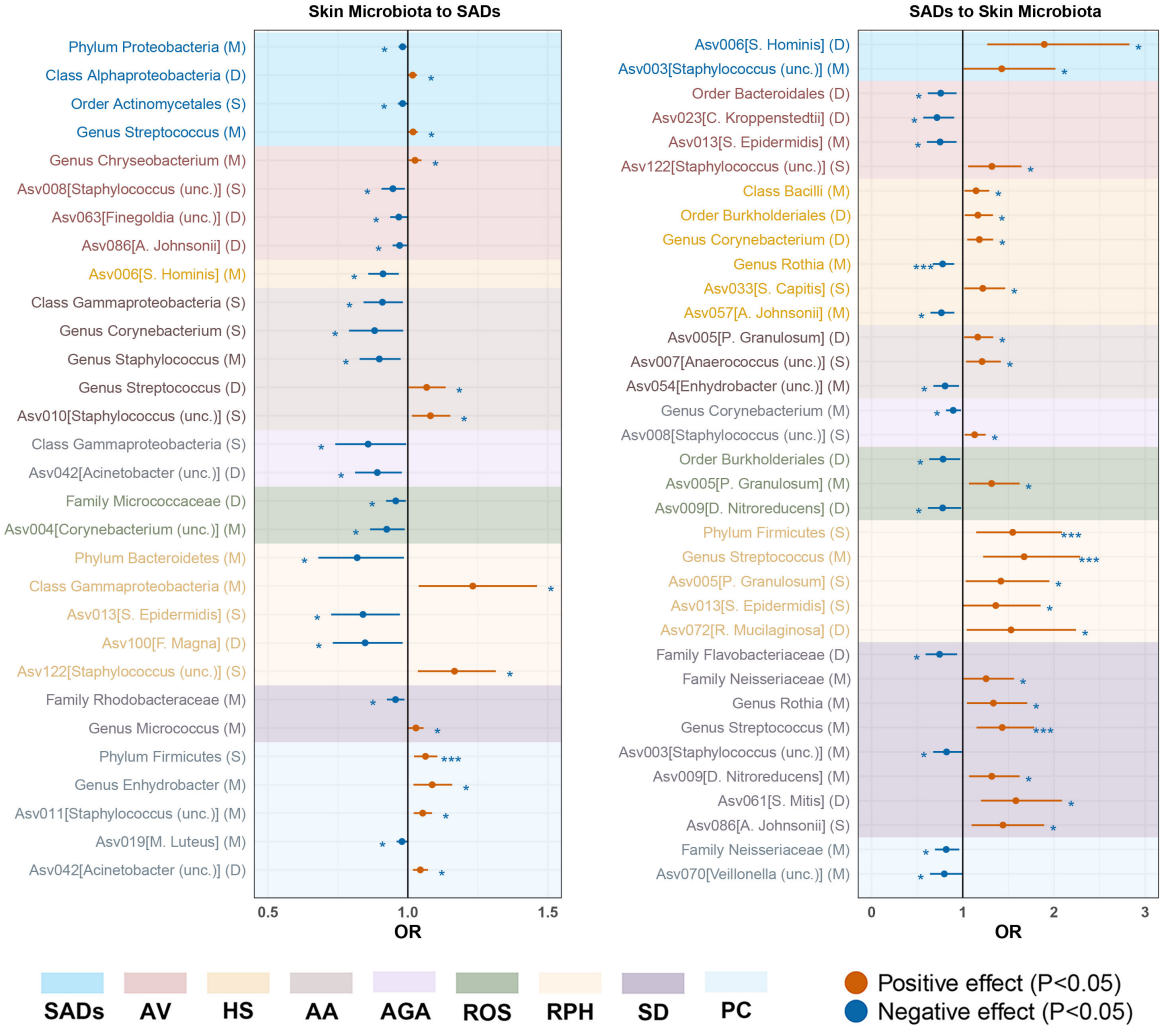
Forest plot of significant bidirectional causalities between skin microbiota and SADs. M, moist; D, dry; S, sebaceous; SADs, skin appendage Disorders; AV, acne vulgaris; HS, hidradenitis suppurativa; AA, alopecia areata; AGA, androgenic alopecia; ROS, rosacea; RPH, rhinophyma; SD, seborrhoeic dermatitis; and PC, pilonidal cyst. * and *** represent nominal causalities and strong causal associations, respectively.

**Figure 4:**
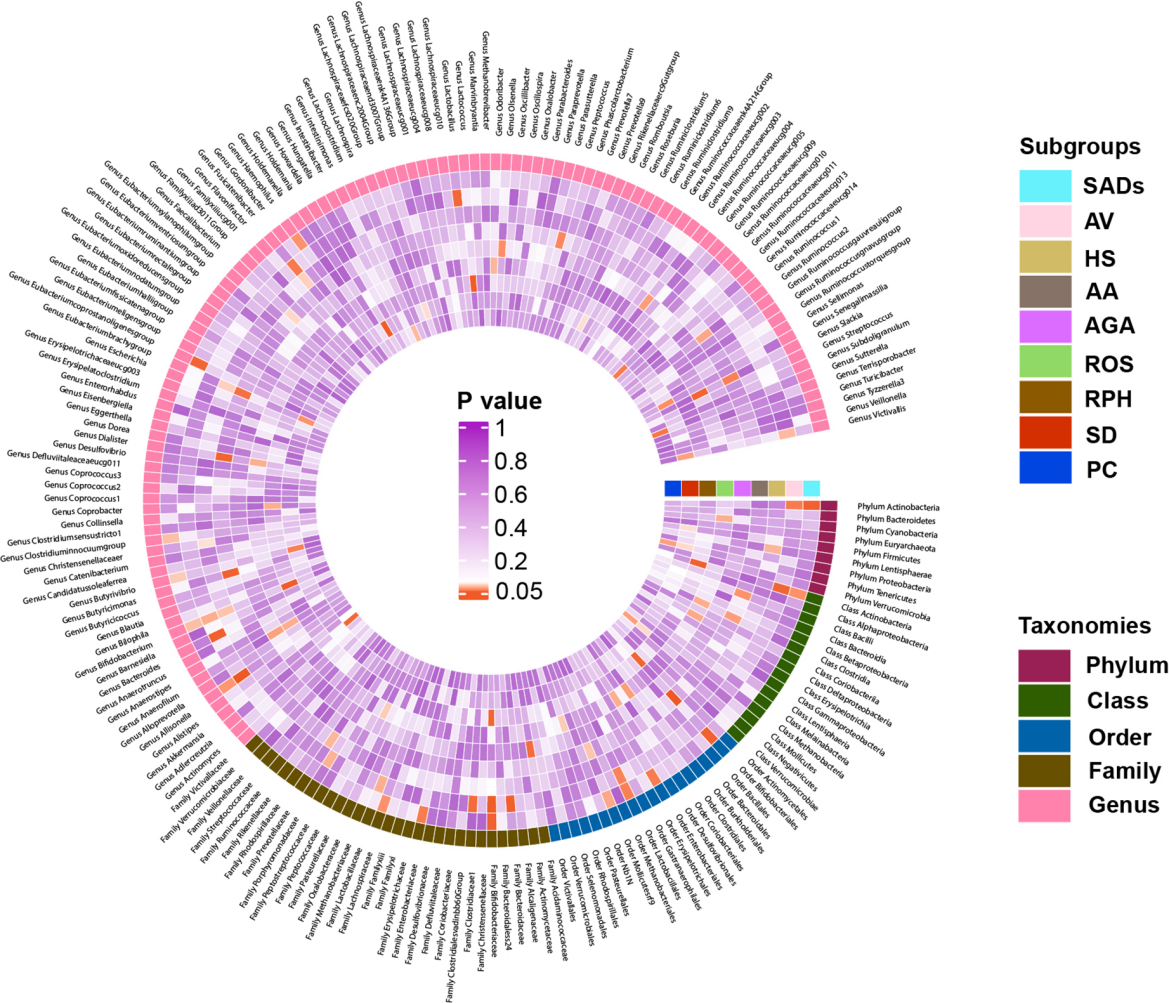
Heatmap showing causal associations between gut microbiota and SADs. SADs, skin appendage Disorders; AV, acne vulgaris; HS, hidradenitis suppurativa; AA, alopecia areata; AGA, androgenic alopecia; ROS, rosacea; RPH, rhinophyma; SD, seborrhoeic dermatitis; and PC, pilonidal cyst.

**Figure 5:**
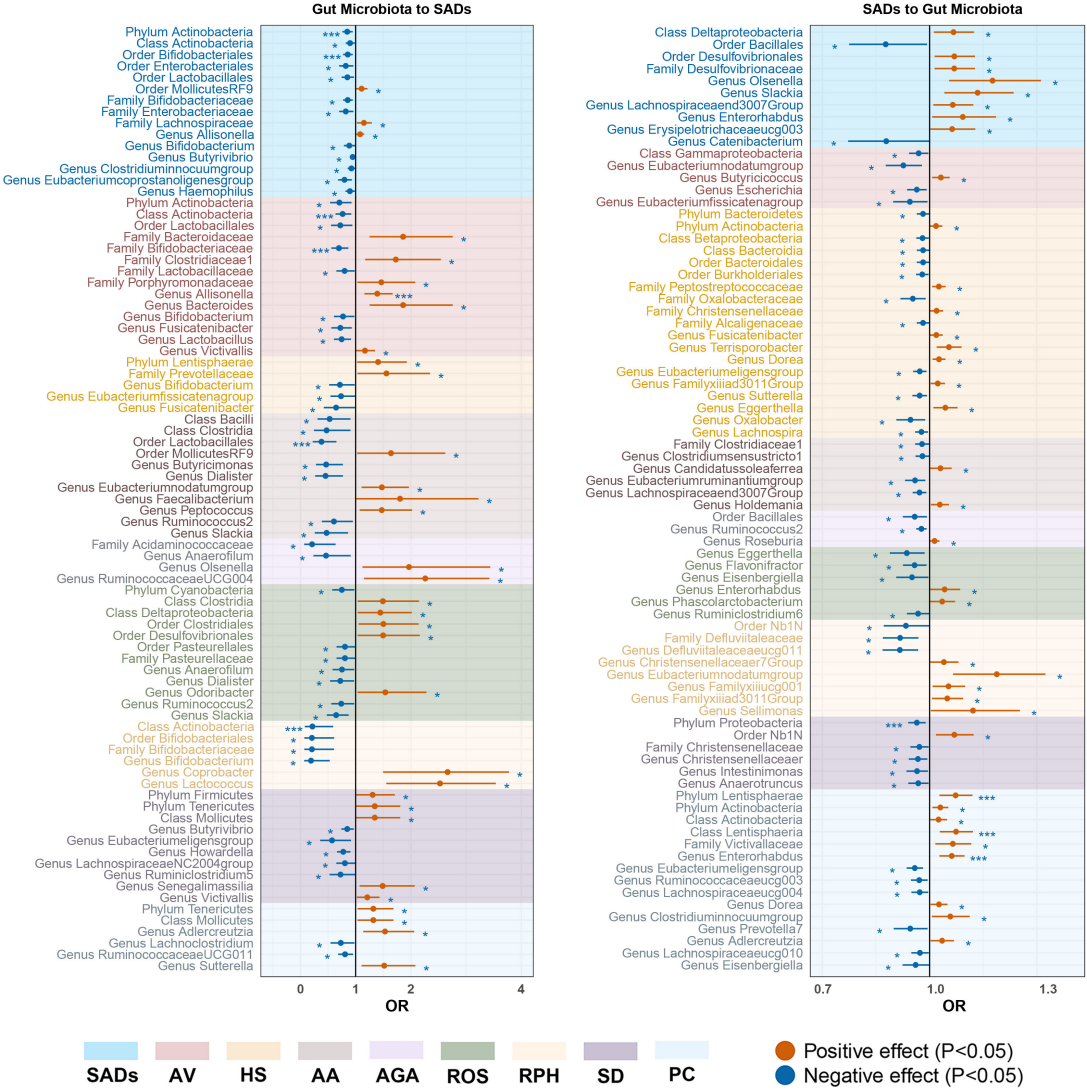
Forest plot of significant bidirectional causalities between gut microbiota and SADs. SADs, skin appendage Disorders; AV, acne vulgaris; HS, hidradenitis suppurativa; AA, alopecia areata; AGA, androgenic alopecia; ROS, rosacea; RPH, rhinophyma; SD, seborrhoeic dermatitis; and PC, pilonidal cyst. * and *** represent nominal causalities and strong causal associations, respectively.

In the forward MR analysis, 115 causal associations were found between SADs and skin microbiota of the KORA FF4 and PopGen cohorts (**Supplementary Table S6**). After the microenvironment-based meta-analysis of two cohorts, 30 causal associations remained significant (*P* < 0.05) in the moist, dry, or sebaceous skin (**Figures 2**, **3**). As for the gut, 83 causal associations were found between SADs and gut microbiota (**Figures 4**, **5**). Details about the causalities between skin and gut microbiota and multiple SADs are shown below.

### SADs

For the skin microbiota, class *Alphaproteobacteria* and genus *Streptococcus* were associated with an inducing effect on total SAD risk. Order *Actinomycetales* and phylum *Proteobacteria* were associated with a protective effect on total SAD risk (**Supplementary Table S8**).

For the gut microbiota, order *Bifidobacteriales* (OR = 0.86, 95% CI = 0.77 - 0.95, *P* = 2.55 × 10^−3^, IVW) and phylum *Actinobacteria* (OR = 0.85, 95% CI = 0.76 - 0.94, *P* = 2.82 × 10^−3^, IVW) displayed strong causal associations with a decreased risk of SADs. Order *MollicutesRF9*, family *Lachnospiraceae*, and genus *Allisonella* were associated with an inducing effect on SADs. Ten bacterial taxa, namely, order *Enterobacteriales* and *Lactobacillales*, class *Actinobacteria*, family *Bifidobacteriaceae* and *Enterobacteriaceae*, and genus *Bifidobacterium*, *Butyrivibrio*, *Clostridiuminnocuumgroup*, *Eubacteriumcoprostanoligenesgroup*, and *Haemophilus*, were associated with a protective effect on SADs risk (**Supplementary Table S10**).

### Acne vulgaris

For the skin microbiota, ASV008 [*Staphylococcus* (unc.)], ASV063 [*Finegoldia* (unc.)] and ASV086 [*A. johnsonii*] were associated with an inducing effect on AV risk. Genus *Chryseobacterium* was associated with a protective effect on AV risk (**Supplementary Table S8**).

For the gut microbiota, class *Actinobacteria* (OR = 0.76, 95% CI = 0.64 - 0.92, *P* = 4.21 × 10^−3^, IVW), and family *Bifidobacteriaceae* (OR = 0.70, 95% CI = 0.56 - 0.87, *P* = 1.08 × 10^−3^, IVW) displayed strong causal associations with a decreased risk of AV. Genus *Allisonella* (OR = 1.39, 95% CI = 1.16 - 1.67, *P* = 3.81 × 10^−3^, IVW) showed a strong causal association with an increased risk of AV. Five bacterial taxa, namely, family *Bacteroidaceae*, *Clostridiaceae1*, *Porphyromonadaceae*, and genus *Bacteroides* and *Victivallis*, were associated with an inducing effect on AV risk. Six bacterial taxa, namely, phylum *Actinobacteria*, order *Lactobacillales*, family *Lactobacillaceae*, and genus *Bifidobacterium*, *Fusicatenibacter*, and *Lactobacillus* were associated with a protective effect on AV risk (**Supplementary Table S10**).

### Hidradenitis suppurativa

For the skin microbiota, ASV006 [*S. hominis*] was associated with a protective effect on the risk of HS (**Supplementary Table S8**).

For the gut microbiota, phylum *Lentisphaerae* and family *Prevotellaceae* were associated with an inducing effect on HS risk. Genus *Bifidobacterium*, *Eubacteriumfissicatenagroup*, and *Fusicatenibacter* were associated with a protective effect on HS risk (**Supplementary Table S10**).

### Alopecia areata

For the skin microbiota, ASV010 [*Staphylococcus* (unc.)] and genus *streptococcus* were associated with an inducing effect on AA risk. Class *Gammaproteobacteria*, genus *Corynebacterium*, and *Staphylococcus* were associated with a protective effect on AA risk (**Supplementary Table S8**).

For the gut microbiota, order *Lactobacillales* (OR = 0.38, 95% CI = 0.22 - 0.65, *P* = 4.07 × 10^−4^, IVW) showed a strong causal association with a decreased risk of AA. Six bacterial taxa, namely, order *MollicutesRF9*, genus *Olsenella*, *RuminococcaceaeUCG004*, *Eubacteriumnodatumgroup*, *Faecalibacterium*, and *Peptococcus*, were associated with an inducing effect on AA risk. Eight bacterial taxa, namely, class *Bacilli* and *Clostridia*, family *Acidaminococcaceae*, and genus *Anaerofilum*, *Butyricimonas*, *Dialister*, *Ruminococcus2*, and *Slackia*, were associated with a protective effect on AA risk (**Supplementary Table S10**).

### Androgenic alopecia

For the skin microbiota, ASV042 [*Acinetobacter* (unc.)] and class *Gammaproteobacteria* were associated with a protective effect on AGA risk (**Supplementary Table S8**).

For the gut microbiota, genus *Olsenella* and *RuminococcaceaeUCG004* were associated with an inducing effect on AGA risk. Family *Acidaminococcaceae* and genus *Anaerofilum* were associated with a protective effect on AGA risk (**Supplementary Table S10**).

### Rosacea

For the skin microbiota, ASV004 [*Corynebacterium* (unc.)] and family *Micrococcaceae* were associated with an inducing effect on ROS risk (**Supplementary Table S8**).

For the gut microbiota, five bacterial taxa, namely, class *Clostridia* and *Deltaproteobacteria*, order *Clostridiales* and *Desulfovibrionales*, and genus *Odoribacter*, were associated with an inducing effect on ROS risk. Phylum *Cyanobacteria*, order *Pasteurellales*, Family *Pasteurellaceae*, and genus *Anaerofilum*, *Dialister*, *Ruminococcus2*, and *Slackia*, were associated with a protective effect on ROS risk (**Supplementary Table S10**).

### Rhinophyma

For the skin microbiota, ASV122 [*Staphylococcus* (unc.)] and class *gammaproteobacteria* were associated with an inducing effect on RPH risk. Phylum *Bacteroidetes*, ASV013 [*S. epidermidis*] and ASV100 [*F. magna*] were associated with a protective effect on RPH risk (**Supplementary Table S8**).

For the gut microbiota, class *Actinobacteria* (OR = 0.22, 95% CI = 0.08 - 0.59, *P* = 2.93 × 10^−3^, IVW) exhibited a strong causal association with a decreased risk of RPH. Genus *Coprobacter* and *Lactococcus* were associated with an inducing effect on RPH risk. Order *Bifidobacteriales*, family *Bifidobacteriaceae*, and genus *Bifidobacterium* were associated with a protective effect on RPH risk (**Supplementary Table S10**).

### Seborrhoeic dermatitis

For the skin microbiota, the genus *Micrococcus* was associated with an inducing effect on SD risk. Family *Rhodobacteraceae* was associated with a protective effect on SD risk (**Supplementary Table S8**).

For the gut microbiota, five bacterial taxa, namely, phylum *Tenericutes* and *Firmicutes*, class *Mollicutes*, and genus *Senegalimassilia* and *Victivallis*, were associated with an inducing effect on SD risk. Genus *Butyrivibrio*, *Eubacteriumeligensgroup*, *Howardella*, *LachnospiraceaeNC2004group*, and *Ruminiclostridium5* were associated with a protective effect on SD risk (**Supplementary Table S10**).

### Pilonidal cyst

For the skin microbiota, phylum *Firmicutes* (OR = 0.22, 95% CI = 0.08 - 0.59, *P* = 2.93 × 10^−3^, IVW) displayed a strong causal association with an increased risk of PC. ASV011[*Staphylococcus* (unc.)], ASV042[*Acinetobacter* (unc.)] and genus *Enhydrobacter* were associated with an inducing effect on PC risk. ASV019[*M. luteus*] was associated with a protective effect on PC risk (**Supplementary Table S8**).

For the gut microbiota, phylum *Tenericutes*, class *Mollicutes*, genus *Adlercreutzia*, and *Sutterella* were associated with an inducing effect on PC risk. Genus *Lachnoclostridium* and *RuminococcaceaeUCG011* were associated with a protective effect on PC risk (**Supplementary Table S10**).

### Reverse MR analysis

In the reverse MR analysis, 119 causal associations were found between SADs and skin microbiota of the KORA FF4 and PopGen cohorts (**Supplementary Table S7**). After the microenvironment-based meta-analysis of two cohorts, 35 causal associations remained significant (*P* < 0.05) in the moist, dry, or sebaceous skin (**Supplementary Table S9**). Among the causalities, HS displayed a strong causal association with a decreased abundance of genus *Rothia* (OR = 0.78, 95% CI = 0.67 - 0.91, *P* = 1.10 × 10^−3^, IVW). RPA showed strong causal associations with an increased abundance of phylum *Firmicutes* (OR = 1.55, 95% CI = 1.14 - 2.08, *P* = 4.20 × 10^−3^, IVW) and genus *Streptococcus* (OR = 1.67, 95% CI = 1.22 - 2.29, *P* = 1.20 × 10^−3^, IVW). SD exhibited a strong causal association with an increased abundance of genus *Streptococcus* (OR = 1.43, 95% CI = 1.15 - 1.78, *P* = 1.30 × 10^−3^, IVW).

As for the gut, 78 causal associations were found between SADs and gut microbiota (**Supplementary Table S11**). Among the causalities, PC showed strong causal associations with an increased abundance of phylum *Lentisphaerae* (OR = 1.07, 95% CI = 1.03 - 1.12, *P* = 1.40 × 10^−3^, IVW), class *Lentisphaeria* (OR = 1.07, 95% CI = 1.03 - 1.12, *P* = 1.10 × 10^−3^, IVW) and genus *Enterorhabdus* (OR = 1.06, 95% CI = 1.03 - 1.10, *P* = 3.50 × 10^−4^, IVW). SD demonstrated a strong causal relationship with a drop abundance in the phylum *Proteobacteria* (OR = 0.96, 95% CI = 0.94 - 0.99, *P* = 4.60 × 10^−3^, IVW).

### Sensitivity analysis

**Figure 6:**
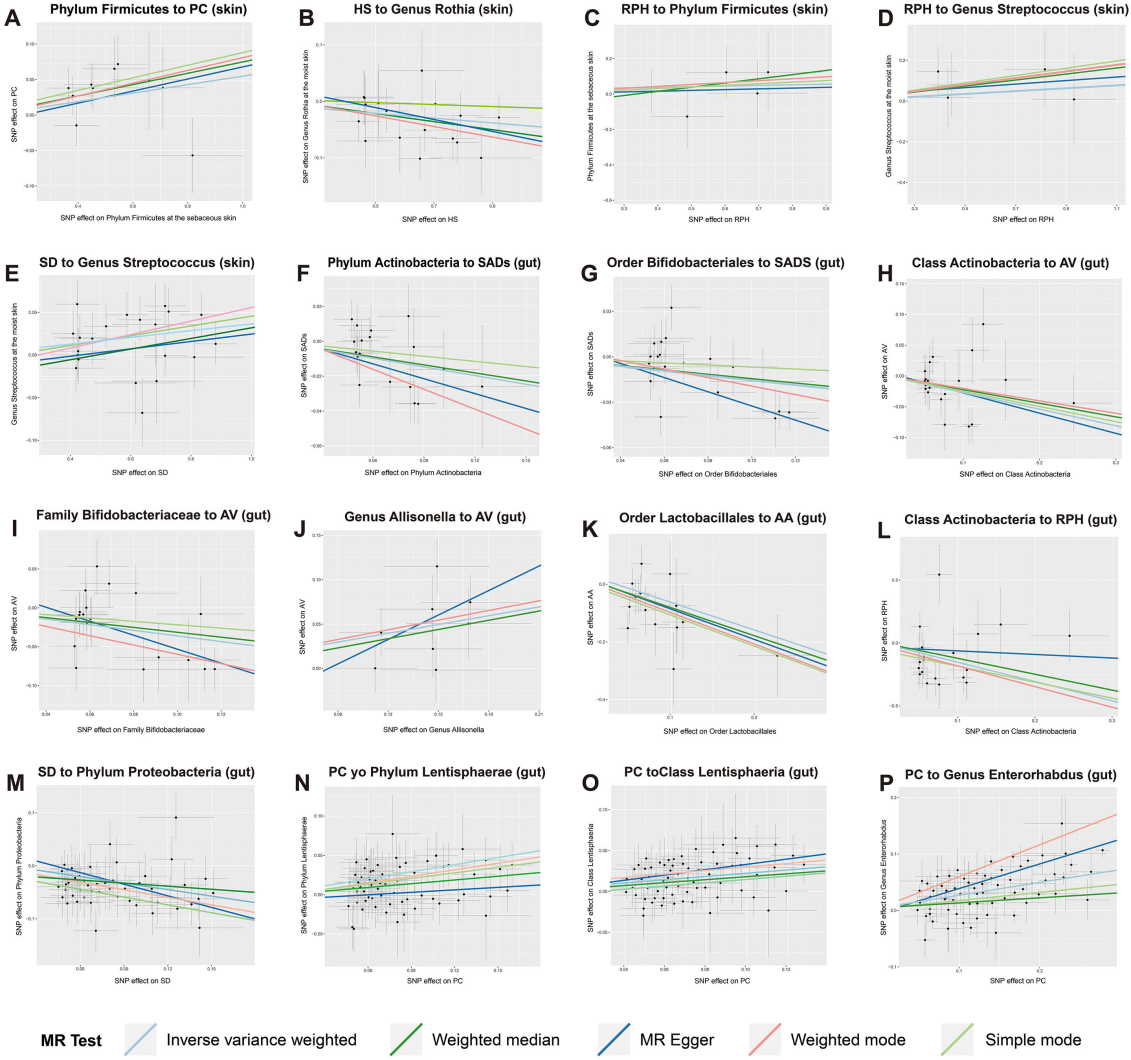
Scatter plots of strong causal associations between skin and gut microbiota and SADs. **(A–E)** the causal associations between skin microbiota and multiple SADs; **(F–P)** the causal associations between gut microbiota and multiple SADs. SADs, skin appendage Disorders; AV, acne vulgaris; SD, seborrhoeic dermatitis; HS, hidradenitis suppurativa; AA, alopecia areata; RPH, rhinophyma; and PC, pilonidal cyst.

The causal estimates for magnitude and direction remained consistent across the weighted median, MR-Egger, weighted mode, and simple mode methods (**Figure 6**). The results of the LOO analysis indicated that no single SNP disproportionately influenced the overall causal estimate. No horizontal pleiotropy of the IVs was detected, as evidenced by the MR-PRESSO global test (*P* > 0.05) and MR-Egger regression (*P* > 0.05). Moreover, the Cochrane *Q* statistics indicated no significant heterogeneity (*P* > 0.05). The Steiger directionality test implied that the causalities identified were free of reverse causality bias (*P* < 0.05) (**Supplementary Table S12-15**).

## Discussion

The results of this study provide a robust foundation for understanding the intricate relationships between the skin and gut microbiota and SADs. A total of 65 and 161 causal relationships between genetic liability in the skin and gut microbiota with SADs were identified, respectively. Among these, we separately found 5 and 11 strong causal associations that passed Bonferroni correction in the skin and gut microbiota with SADs. Several skin bacteria, such as *Staphylococcus*, *Streptococcus*, and *Propionibacterium*, were considered associated with multiple SADs. As gut probiotics, *Bifidobacteria* and *Lactobacilli* were associated with a protective effect on SAD risk. Our findings indicated the crucial role that gut and skin microbiota play in the host-microbiota interaction of skin diseases, supporting the notion that adjusting the host-microbe balance is crucial for the prevention and therapy of SADs.

Commensal bacteria are crucial for maintaining the immune system. Numerous studies have investigated the gut microbiome concerning various illnesses, including its effects on the skin (5). Skin bacteria, although less numerous than those found in the gastrointestinal tract, have comparable roles in immunological modulation and disease development (9). It is increasingly evident that both the cutaneous and gut microbiomes profoundly impact human health, particularly in the context of SADs.

*P. acnes* is recognized as the main disease-associated bacterium in the case of AV (30, 31). Research comparing the skin of acne sufferers and healthy individuals found that while the relative abundances of *P. acnes* species were similar, significant differences were observed at the strain level between the two cohorts (32). Certain strains had a strong connection to acne, whereas other strains were more prevalent in healthy skin. Interestingly, microbiome research has shown that AV is more closely associated with the virulence of specific *P. acnes* strains rather than the total quantity of *P. acnes* (31). Dysbiosis in AV is indicated by a reduction in the percentage of *P. acnes* strains RT6 and a notable increase in the percentages of *P. acnes* strains RT4, RT5, RT7, RT8, RT9, and RT10 (33). Moreover, a competitive relationship has been observed between *S. epidermidis* and *C. acnes* (34, 35). *S. epidermidis* promotes glycerol fermentation and releases succinic acid, which prevents *C. acnes* from proliferating (34). Conversely, *P. acnes* maintains the acidic environment of the pilosebaceous follicle, hydrolyzes sebaceous triglycerides, and produces propionic acid to inhibit *S. epidermidis* proliferation (35). Our research demonstrated that ASV001 [*P. acnes*] at sebaceous skin sites was positively correlated with AV in the PopGen cohort, whereas AV was negatively correlated with ASV013 [*S. epidermidis*] at moist skin sites in the KORA FF4 cohort. Although these associations lost significance in the meta-analysis, they potentially illustrate the role of *P. acnes* in promoting acne and inhibiting the colonization of *S. epidermidis* in acne patients.

*Bifidobacteria* and *Lactobacilli*, the commensal microorganisms inhabiting the gastrointestinal tract, have garnered interest for their therapeutic potential as probiotics in the amelioration of inflammatory dermatological conditions, including AV (34, 36). Their therapeutic actions are purportedly mediated through the modulation of systemic oxidative stress levels, the regulation of cytokine production, and the attenuation of inflammatory biomarkers (34). Clinical investigations have revealed that the administration of a composite probiotic formulation containing *Lactobacillus acidophilus*, *Lactobacillus delbrueckii bulgaricus*, and *Bifidobacterium bifidum* may rival the efficacy of conventional antibiotic therapy, such as minocycline, in acne management (37). An observed lesion reduction of 67% after a 12-week therapeutic regimen, coupled with a reduced incidence of adverse effects, substantiates this claim (37). The oral administration of various *Lactobacillus* species has been shown to reduce the total lesion count by 56%-67%, decrease sebum content by 81%, and improve the Investigators Global Assessment in 80% of patients (38). The MR outcomes of our research are consistent with the findings of previous studies. *Bifidobacteria* exhibited a significant causal association with inhibiting AV, and *Lactobacilli* were also regarded as potential protective factors for AV. Notably, the genus *Allisonella* showed a significant causal effect on facilitating AV, which may be due to its unique biochemical capability to produce histamine, a compound associated with gut inflammation, immune response, and allergic reactions (39).

Microbial changes in patients with AA revealed a lower abundance of *S. epidermidis* and over-colonization with *P. acnes*; however, it remains unclear if these alterations are the cause or effect of the illness (35). In this study, AA was positively correlated with ASV005 [*P. granulosum*] at moist skin sites, while the genus *Staphylococcus* at moist skin sites displayed a potential inhibitory effect on AA. Recent research has also connected AA to gut dysbiosis in addition to skin microbiota alterations. The role of the gut microbiome in the pathogenesis of AA is supported by cases of long-term hair growth following fecal microbiota transplants (40). However, some detected variations in gut flora in AA patients were not statistically significant (41). Following the Bonferroni adjustment, no apparent causal relationships between AA and the gut microbiota were found according to the reverse MR analysis, which aligns with previous studies. However, this MR study indicated that the order *Lactobacillales* displayed a significant causal association with inhibiting AA. Mechanistic studies have shown that *Lactobacillales*, as an important probiotic group, may influence AA through the modulation of immune function (36). *Lactobacillales* can regulate the body’s immune response via various mechanisms, such as promoting the production of regulatory T cells and reducing the release of pro-inflammatory cytokines (42). This immunomodulatory action may contribute to the suppression of the autoimmune response underlying AA.

There is growing evidence that a dysbiotic skin microbiome is associated with HS (43); however, the exact causal link between changes in the skin microbiome and the onset of the disease is still unknown. It was found that the bacterial community on the skin surface of HS patients was significantly altered, primarily characterized by a significant decrease in *Staphylococcus epidermidis* and *Staphylococcus hominis* in the axilla, gluteal cleft, and groin areas of HS patients (44). *Propionibacterium* was also observed to be more abundant in controls than in HS patients (44). The MR outcomes indicated that ASV006 [*S. hominis*] at moist skin sites was regarded as a potential protective factor for HS, and HS could induce the growth of ASV033 [*S. capitis*] at sebaceous skin sites. However, no causal association was found between *Propionibacterium* and HS, suggesting that this genus may not have a direct impact on the etiology of the illness. This dysbiosis of the skin microbiome may be related to chronic inflammation and a hypoxic environment in the HS focal areas. There are several comorbid conditions linked to HS. Notably, patients with HS have up to eight times higher rates of inflammatory bowel disease compared to the general population, with Crohn’s disease outpacing ulcerative colitis in frequency (45). An altered gut microbiota may be a factor in the development of HS since it has been linked to several pathophysiologies, including immune dysregulation (46). In this study, the genera *Bifidobacterium*, *Eubacterium fissicatenagroup*, and *Fusicatenibacter* exhibited potential causal effects on inhibiting HS, while the family *Prevotellaceae* and phylum *Lentisphaerae* showed potential causal effects on promoting HS. However, the specific mechanisms and modes of intervention for these causalities need to be further explored.

ROS, RPH, and SD are skin conditions associated with microbiological and immunological dysbiosis of the skin environment, as well as with *Demodex mites* and *Malassezia fungus* (47–49). Microbiota-associated alterations in the skin and small intestine have been concurrently noted in these diseases. Since ROS is exacerbated or triggered by emotional stress, the brain may play a role in the gut-brain-skin axis (47, 48). Unfortunately, conflicting findings have been reported in microbial research conducted on ROS patients at both the skin and gut levels (50). Some bacteria thought to be connected to ROS include *S. epidermidis*, *Helicobacter pylori*, *Chlamydophila pneumoniae*, and *Bacillus oleronius* (50). Our MR results indicated that ASV013 [*S. epidermidis*] at sebaceous skin sites could reduce the incidence of RPH, which is often considered a late complication of ROS. It has been suggested that ROS may be improved by taking oral probiotics such as *Lactobacillus salivarius* and *Bifidobacterium*, which was also observed in the MR results (36). The family *Bifidobacteriaceae* and the genus *Bifidobacterium* exhibited potential causal associations with inhibiting RPH. Additionally, it was reported that *Acinetobacter*, *Staphylococcus*, and *Streptococcus* predominated in lesional skin when examining the bacterial microbiota in 24 individuals with SD (3). This phenomenon was also reflected in the reverse MR analysis, which showed an increase in the genus *Streptococcus* and ASV086 [*A. johnsonii*]. Furthermore, it was shown that the phylum *Firmicutes* significantly contributed to the development of PC. Nevertheless, the connection between PC and the skin and gut microbiota has not been well studied, and further research is still required.

The intricate relationships between the skin and gut microbiota and SADs can be explained through several biological mechanisms. Firstly, microbes can significantly influence the host immune system. For instance, *Staphylococcus aureus* is known to produce superantigens and other virulence factors that hyperactivate the immune system, leading to chronic inflammation and tissue damage observed in conditions such as atopic dermatitis and hidradenitis suppurativa (51). These virulence factors can trigger the release of pro-inflammatory cytokines such as IL-1β, IL-6, and TNF-α, contributing to the inflammatory milieu characteristic of these disorders (52). Conversely, beneficial gut microbes like *Bifidobacteria* and *Lactobacilli* play a protective role by modulating systemic immune responses. These probiotics can enhance the production of anti-inflammatory cytokines, such as IL-10, and promote the differentiation of regulatory T cells, which help maintain immune homeostasis and prevent excessive inflammation (53). Secondly, microbial metabolites, such as short-chain fatty acids (SCFAs), play a crucial role in maintaining skin health. SCFAs like butyrate, acetate, and propionate, produced by gut microbiota during the fermentation of dietary fibers, have potent anti-inflammatory properties and can strengthen gut barrier function (54). These metabolites can enter the bloodstream and exert systemic effects, including on the skin. For example, SCFAs have been shown to enhance the differentiation of keratinocytes and promote wound healing, which could be beneficial for conditions like eczema and psoriasis (55). Moreover, the integrity of epithelial barriers in both the gut and skin is vital for preventing pathogen invasion and maintaining overall health. In the gut, harmful bacteria can disrupt tight junction proteins, leading to increased intestinal permeability, also known as “leaky gut,” which allows endotoxins to enter the bloodstream and trigger systemic inflammation (56). This systemic inflammation can adversely affect skin health, exacerbating conditions like acne and psoriasis (57).

Although the conservativeness of the Bonferroni correction method may lead to false negatives (58), the stringent control provided by the Bonferroni correction is essential for the integrity of our findings, thereby enhancing the reliability of causal inferences in genetic epidemiology (27, 28, 59). Many causal correlations in this study were regarded as nominally significant due to failing the Bonferroni corrected test. We speculate that the contribution of a single microbiome to illness may not be as substantial as initially estimated. Rather, the illnesses may be caused and coordinated by multiple bacteria. These bacteria with nominal causalities may also be involved in skin and intestinal-related SADs. The investigation of the human microbiota in SADs is presently ongoing, and the precise role of the microbiome in the pathophysiology of SADs remains to be thoroughly studied. Understanding the causality of the interplay between various microbiotas and SADs can aid in comprehending the intricate crosstalk between the skin and gut, and offer guidance for future targeted multi-flora medication development.

To the best of our knowledge, this is the first MR study to comprehensively examine the causal effect between skin and gut microbiota and SADs. A bidirectional, two-sample MR design following STROBE-MR guidelines was employed to eliminate the potential for reverse causation and confounding factors. Exposure and outcome summary data were separately acquired from German and Finnish populations to ensure nonoverlapping data sets and avoid bias. A microenvironment-based meta-analysis of skin microbiota was conducted to enhance the statistical power of the results. However, several limitations of our study should be noted. First, we included SNPs that met the locus-wide significance level (1 × 10^−5^), as the SNPs identified using the genome-wide significance threshold (5 × 10^−8^) were insufficient for sensitivity analysis and horizontal pleiotropy detection. Second, some taxa of skin microbiota at the ASV level lacked species-level annotations, possibly due to uncertain matches to the Ribosomal Database Project (RDP) database. Additionally, most 16S rRNA sequencing of human microbiota has focused on species composition. Recent research has indicated that distinct strains of microorganisms can exert significantly different effects on the host, even within the same species (60). Variations at the strain level have not been well studied and remain an area of interest for microbiota research. Third, there is limited knowledge of the causal link between SADs and non-bacterial components of the microbiota, such as fungi, archaea, and viruses. Therefore, future research using more advanced sequencing technology should be conducted to further elucidate the effects of skin and gut microbiota on SADs.

In summary, our study provided comprehensive evidence for the causal roles of skin and gut microbiota in SADs through the application of bidirectional MR analysis. This novel approach allowed us to establish strong causal links between specific microbial taxa and various SADs, underscoring the intricate interplay between microbial communities and skin health. The identification of specific skin and gut microbiota that influence the risk of SADs offers promising avenues for the development of microbiome-based diagnostic and therapeutic strategies. Future studies involving genomic, transcriptomic, and metabolomic analyses should focus on elucidating the underlying mechanisms of how specific microbes influence SADs and modulate skin health.

## Data Availability

The datasets presented in this study can be found in online repositories. The names of the repository/repositories and accession number(s) can be found in the article/**Supplementary Material**.

## Supplementary material

Additional File 2 STROBE-MR checklist of suggested items for Mendelian randomization studies.
